# Left atrial appendage morphology and risk of stroke following pulmonary vein isolation for drug-refractory atrial fibrillation in low CHA_2_DS_2_Vasc risk patients

**DOI:** 10.1186/s12872-017-0504-7

**Published:** 2017-02-28

**Authors:** Faith R. Kelly, Robert A. Hull, Takor B. Arrey-Mbi, Michael U. Williams, Joshua S. Lee, Ahmad M. Slim, Dustin M. Thomas

**Affiliations:** 1 Cardiology Department, Brooke Army Medical Center, 3551 Roger Brooke Drive, San Antonio, TX 78234-6200 USA; 2 Department of Medicine, Brooke Army Medical Center, San Antonio, TX USA

**Keywords:** Left atrial appendage, Stroke, CVA, Pulmonary vein isolation, PVI, Atrial fibrillation, CHA2DS2Vasc, Cardiac CT Angiography, CCT

## Abstract

**Background:**

Cardiac CT angiography (CCTA) has become an important adjunct in the structural assessment of the pulmonary veins (PV) prior to pulmonary vein isolation (PVI). Published data is conflicting regarding a relationship between left atrial appendage (LAA) and the risk of ischemic stroke (CVA) following PVI. We investigated the associations of volumetric and morphologic left atrial (LA) and LAA measurements for CVA following PVI.

**Methods:**

We retrospectively reviewed 332 consecutive patients with drug refractory atrial fibrillation who obtained cardiac CT angiogram (CCTA) prior to PVI. Baseline demographic data, procedural and lab details, and outcomes were obtained from abstraction of an electronic medical records system. LA, LAA, and PV volumes were measured using CCTA datasets utilizing a semi-automated 3D workstation application. LAA morphology was assigned utilizing volume rendered images as previously described.

**Results:**

The study cohort was 55 ± 13 years-old, 83.7% male, low CVA risk (median CHA_2_DS_2_Vasc 1; IQR 1, 3), and 30.4% were treated with novel oral anticoagulants. Chicken wing (CW) was the most common morphology (52%), followed by windsock (WS), cauliflower (CF), and cactus (CS) at 18, 9, and 2%, respectively. CVAs occurred in 4 patients following PVI with median time to CVA of 170.5 days. All CVAs were observed in CW morphology patients. When comparing CW morphology with non-CW morphology, CVAs occurred more frequently with the CW morphology (2.1% vs 0%, *p* = 0.03). This difference was not significant, though, after adjusting for CHA_2_DS_2_Vasc risk factors (*p* = 0.14).

**Conclusion:**

The CW morphology was observed more commonly in patients who experienced post-PVI CVA. After adjusting for CHA_2_DS_2_Vasc risk factors, CW morphology was not an independent predictor of post-PVI CVA. These findings should be interpreted in the setting of a low CVA event rate amongst a low risk population that was highly compliant with indicated anticoagulation therapy.

## Background

Atrial fibrillation (AF) is an increasingly prevalent disease with significant associated morbidity and mortality, principally due to cerebral vascular accident (CVA) and from symptomatic arrhythmia [1]. Pulmonary vein isolation (PVI) continues to emerge as an effective treatment for symptomatic AF [2, 3]. Cardiac CT angiography (CCTA) has become an important adjunct in the structural assessment of the pulmonary veins prior to PVI [4–6]. Additionally, the left atrial appendage (LAA), a common nidus of thrombus formation in AF patients, is well visualized and can be evaluated for the presence of thrombus in appropriately protocoled studies [7–9]. Published data suggests an association between LAA morphology, as determined by CCTA, and risk for CVA in patients referred for PVI [10]. In the largest of these studies, the chicken wing (CW) morphology was 66% less likely to be present in patients with a history of CVA, whereas the cactus morphology was the highest risk LAA morphology [10]. The data on LAA morphology and predicting CVA following PVI is less robust. We sought to determine CCTA-derived factors associated with CVA in patients who underwent PVI for drug-refractory AF in a single-center military hospital.

## Methods

### Population

**Fig. 1:**
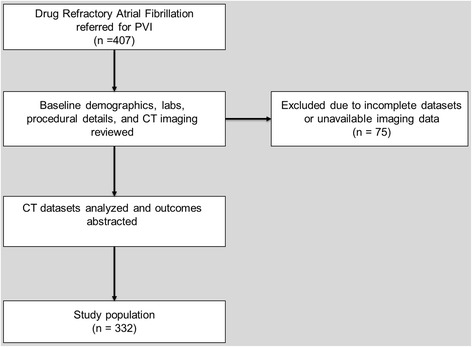
Flow chart depicting patient screening and reasons for exclusion from final analysis

We identified 407 patients with drug-refractory atrial fibrillation who underwent PVI between January 2008 and December 2014 at large military treatment facility. Baseline demographic data, procedural and lab details, and outcomes were obtained from manual chart abstraction of the electronic medical records (EMR). Patients with incomplete or unavailable imaging data were excluded from analysis (Fig. 1). Hypertension was defined as previous diagnosis, currently taking any anti-hypertensive medications, or a recorded systolic blood pressure > 140 mm Hg on 2 separate encounters. Congestive heart failure was defined as a previous diagnosis based on review of past medical history. Diabetes mellitus was defined as a previous diagnosis based on EMR review, currently taking any anti-hyperglycemic medications, or a most recent hemoglobin A1c value ≥ 6.5%. Vascular disease was defined as any previous history of myocardial infarction, coronary revascularization (percutaneous coronary intervention or coronary artery bypass grafting), or peripheral arterial disease. CVA was identified by EMR review and adjudicated utilizing the inpatient EMR or assessment by an outpatient neurologist evaluation. These risk factors, in addition to age and gender, were used to calculate a CHA_2_DS_2_Vasc score [11]. Post-PVI anticoagulation was recorded as the oral systemic agent on which the patient was discharged following PVI. Choice of oral systemic agent was at the discretion of the electrophysiologist performing the PVI. Post-PVI CVA events were identified utilizing the same criteria as outlined above. Patients with TIA by ICD-9 code listed following PVI were reviewed, but these instances were excluded unless deemed by the investigators to represent an embolic event. The vast majority of patients underwent pre-procedural transesophageal echocardiograph (89.5%) with finding of possible thrombus in one patient. CCTA in this patient performed with early and delayed phases was able to exclude thrombus. As the vast majority of pre-procedural CCTAs were obtained with only a single, early phase, all patients with LAA filling defects by CCTA were excluded from this analysis.

### Pulmonary Vein Isolation (PVI)

All PVIs were performed utilizing bilateral femoral venous access. For patients on warfarin therapy, this was continued without bridging anticoagulation in the peri-procedural period. In patients on NOACs leading up to PVI, these medications were held for 24 h prior to the procedure. Regardless of pre-procedural systemic anticoagulation agent, intravenous heparin was administered with a goal ACT of ≥350 s. ACT readings were reassessed at 30 min intervals and additional heparin boluses were administered as needed while catheters were in the left atrium. The esophagus was marked with a temperature probe and intracardiac echocardiography (ICE) was utilized for transseptal puncture. Transseptal puncture was performed using Brockenbrough needle under fluoroscopic and ICE guidance. Left atrial access was confirmed utilizing pressure transduction and saline administration. A multipolar mapping catheter was placed into the left atrium at operator discretion. Electroanatomic 3-D mapping of the left atrium was performed. Using contact force mapping, lesions were placed in a circumferential manner around the pulmonary veins. All ablation lesions were performed utilizing an open-irrigated catheter ablation system. The possibility of esophageal injury was minimized by performing ablation at < 30 watts along the posterior wall and terminating ablation with detected temperature rise in the esophageal probe. All other ablation lesions were performed utilizing 30–40 watts. Pacing at the right superior pulmonary vein was performed to confirm the absence of diaphragm stimulation prior to ablation at operator’s discretion. A multi-polar mapping catheter was then used to assess for any gaps. Immediate post-ablation isolation of the pulmonary veins was confirmed by LA and PV pacing to ensure bidirectional block. Non-PV triggers were induced utilizing isoproterenol and additional radiofrequency ablations administered as indicated. The ablation catheter was withdrawn to the right atrium. Screening for pericardial effusion was performed at the end of the case and pulmonary vein stenosis excluded using ICE. All catheters were then removed and, after reversal of anticoagulation with protamine, all sheaths were removed. All patients, regardless of CHA_2_DS_2_Vasc score, received 4 weeks of systemic anticoagulation (warfarin or a NOAC) following PVI. Choice of anticoagulant was at the discretion of the electrophysiologist performing the PVI.

### Cardiac computed tomography

**Fig. 2:**
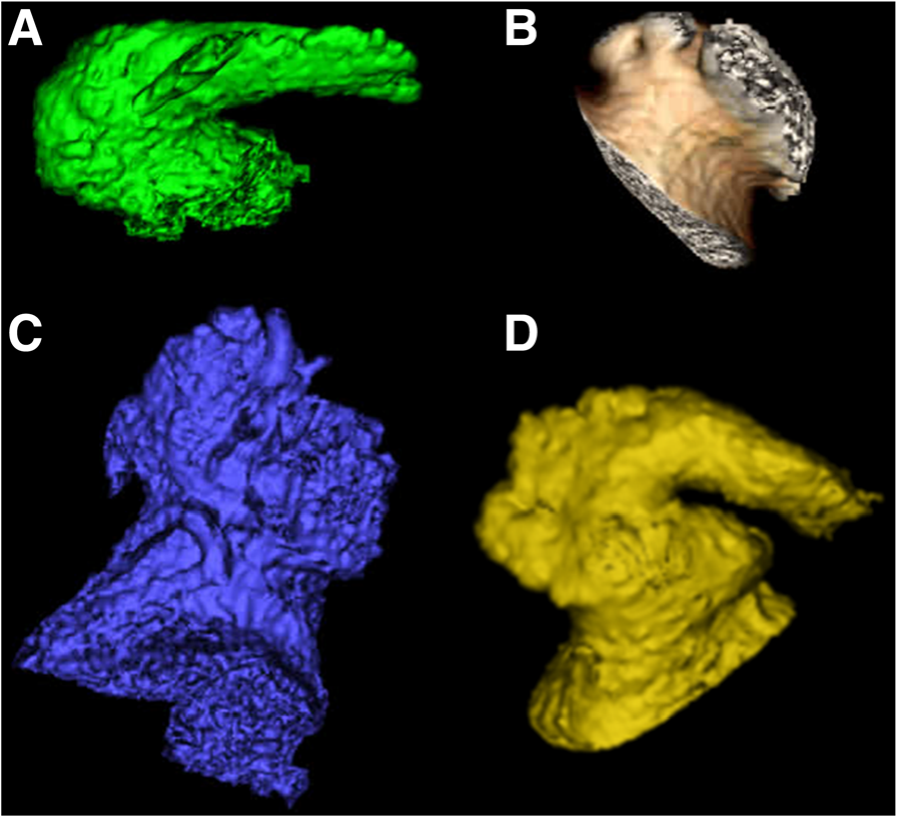
Volume rendered cardiac CT angiography images demonstrating chicken wing (**a**), cauliflower (**b**), cactus (**c**), and windsock (**d**) morphologies

From January of 2008 to March of 2011, images were obtained using a retrospective ECG-gated acquisition with a 64-slice CT scanner (Somatom Definition CT, Siemens, Erlangen, Germany) reconstructed at 0.7 mm slices centered on the 70% R-R interval. From March of 2011 to March of 2012, CCTA was performed utilizing a prospective ECG-triggered acquisition centered on the 60 ± 20% R-R interval. After March of 2012, a 128-slice dual-source scanner (Somatom Definition Flash CT, Siemens, Erlangen, Germany) utilizing prospective, ECG-triggered acquisition centered on the 60 ± 20% R-R interval was used. CCTA images were evaluated on a 3-dimension workstation (Vital Images, Inc., Minnetonka, MN). Left atrial (LA) and LAA parameters were obtained using electrophysiology planning software package. Manual segmentation of the LA and LAA was performed by delineating the borders utilizing axial reconstructions. LAA volumes were included in the LA volumes, however PV volumes were excluded. The LAA os was pre-determined to be the proximal border of the LAA and all visualized lobes were included in the volumes. LAA morphology (Fig. 2) was assigned by a single investigator (FK) based on review of volume rendered reconstructions and reported as chicken wing (CW), cauliflower (CF), cactus (CS), and windsock (WS) as previously described [10]. The CW was defined as a LAA morphology with a discrete bend in the proximal to mid-portion of the dominant lobe, folding back upon itself at some distance from the LAA ostium10. This morphology could also contain secondary lobes or twigs. The CF morphology was considered when LAA had a limited overall length and more complex internal characteristics. It was distinguished from other morphologies by variations in the ostium shape (oval vs round) and a variable number of lobes without an identifiable dominant lobe [10]. The CS morphology was classified as a dominant central lobe and secondary lobes that extend in both a superior and inferior direction [10]. Finally, the WS morphology was defined as a dominant lobe of sufficient length as the primary structure. Variations on this description to include different locations and numbers of smaller, secondary or even tertiary lobes were included in this morphologic group [10].

### Statistical analysis

Statistical analysis was performed using IBM SPSS version 19.0 (IBM, Armonk, NY). Parametrically distributed continuous variables are reported as means with standard deviation and nonparametric data are reported as medians with interquartile range. Comparison between continuous variables was performed utilizing a Chi squared test or a Wilcoxon Rank sum test, as appropriate. Cutoff values with sensitivity and specificity were performed using a receiver operator characteristics (ROC) curve. Multivariate logistic regression was performed to assess for independent CVA predictors. A Bonferroni correction was applied when appropriate. A cutoff of *p* < 0.05 was used to represent statistical significance. This research was carried out with the approval of our local institutional review board (IRB).

## Results

The patient population of 332 individuals (mean age 55 ± 13 years, 83.7% male) were followed for median of 72 months. Table 1 summarizes baseline demographic data in patients with CW compared with non-CW LAA morphology. No difference was observed in CHA_2_DS_2_Vasc scores between CW (median = 1 [IQR 1, 2]) and non-CW (median = 2 [IQR 1, 4]) patients (*p* = 0.702). Anticoagulation for CVA prophylaxis was achieved with a novel oral anticoagulant in 30.4% of patients and warfarin (pre-procedural INR 2.2 ± 0.5) in 69.6% (*p* < 0.001). There was no difference observed between median proximate INR in warfarin-treated patients (*p* = 0.432) and presenting rhythms (sinus rhythm, atrial arrhythmia, or paced) prior to PVI were similar between CW and non-CW patients.

**Table 1:** Baseline demographic data in chicken wing (CW) morphology patients compared with non-CW patients. All continuous variables are displayed as mean values ± standard deviation unless otherwise annotated

	Total	CW	Non-CW	*p*-value
	(*n* = 332)	(*n* = 190)	(*n* = 142)	
Age	55 ± 13	54 ± 13	56 ± 13	0.263
Age ≥ 75 years	13 (15.2%)	5 (12.5%)	8 (5.6%)	0.490
Male gender	278 (83.7%)	160 (84.2%)	118 (83.1%)	0.785
HTN	200 (60.2%)	115 (60.5%)	85 (59.9%)	0.902
CHF	22 (6.6%)	14 (7.4%)	8 (5.6%)	0.518
DM2	48 (14.5%)	26 (13.7%)	22 (15.5%)	0.644
Vascular disease	140 (42.2%)	79 (41.6%)	61 (43.0%)	0.801
CHA_2_DS_2_Vasc > 1	162 (48.8%)	86 (45.3%)	76 (53.5%)	0.136
CHA_2_DS_2_Vasc, median (IQR)	1 (1, 3)	1 (1, 2)	2 (1, 4)	0.702
Prior CVA/TIA	16 (4.8%)	9 (4.7%)	7 (4.9%)	0.935
Post-PVI CVA	4 (1.2%)	4 (2.1%)	0 (0.0%)	0.034
Anticoagulation				
Warfarin	201 (69.6%)	123 (73.7%)	78 (63.9%)	0.055
NOAC	88 (30.4%)	44 (26.3%)	44 (36.1%)	0.055
Aspirin	43 (13.0%)	23 (12.1%)	20 (14.1%)	0.589
Pre-procedure INR^a^	2.2 ± 0.5	2.2 ± 0.5	2.2 ± 0.5	0.432
Rhythm prior to PVI				
Sinus rhythm	161 (48.8%)	88 (46.8%)	73 (51.4%)	0.407
Atrial Arrhythmia	166 (50.3%)	97 (51.6%)	69 (48.6%)	0.589
Paced	3 (0.9%)	3 (1.6%)	0 (0%)	0.131
Follow-up, mo., median (IQR)	72 (49, 97)	73 (49, 107)	69 (50, 92)	0.344

*CW* chicken wing, *HTN* hypertension, *CHF* congestive heart failure, *DM2* diabetes mellitus, type 2, *CVA* cerebral vascular accident/stroke, *TIA* transient ischemic attack, *PVI* pulmonary vein isolation, *NOAC* novel oral anticoagulant, *INR* international normalized ratio

^a^Only includes patients treated with warfarin at the time of pulmonary vein isolation (PVI)

The overall prevalence of post-PVl CVA was 1.2% (*n* = 4) with median time to CVA of 170.5 days. In patients with post-PVI CVA, the CHA_2_DS_2_Vasc score was zero in one patient, 1 in two patients, and 2 in the fourth patient. Three patients were taking warfarin (one with a sub-therapeutic INR at the time of CVA) and one patient was treated with dabigatran. Post-PVI CVA was observed more frequently in patients with CW morphology compared to non-CW morphologies (2.1% vs 0%, *p* = 0.03). After adjusting for known CVA risk factors, this difference no longer met threshold for statistical significance (*p* = 0.14). There was no difference between the groups with respect to the prevalence of pre-PVI CVA (4.7% vs 4.9%, *p* = 0.935). CHA_2_DS_2_Vasc score was predictive of pre-PVI CVA events (AUC 0.799, *p* < 0.001), but was not discriminatory with respect to predicting post-PVI CVA over 72 months of follow-up (AUC 0.309, *p* = 0.188).

Measured LAA volumes, LA volumes, and average Hounsfield unit (HU) attenuation values in CW and non-CW morphologies are listed in Table 2. No difference was observed between the absolute volume measures between the groups, nor between volume and HU attenuation ratios between the two groups. When comparing patients with post-PVI CVAs to the rest of the population (Table 3), there was also no difference between LAA HU attenuation or the ratio of LAA:LA HU attenuation as can be seen in the setting of spontaneous echocontrast or thrombus.

**Table 2:** Volumetric and CT attenuation data in chicken wing (CW) morphology patients compared with non-CW patients

CT Characteristics	Total	CW	Non-CW	*p*-value
	(*n* = 332)	(*n* = 190)	(*n* = 142)	
LAA volume	13.8 ± 6.0	13.6 ± 5.6	14.1 ± 6.4	0.402
LA volume	99.5 ± 33.6	99.8 ± 34.5	99.2 ± 32.6	0.871
LAA/LA volume	0.145 ± 0.06	0.143 ± 0.05	0.148 ± 0.06	0.454
LAA HU	342.2 ± 77.9	340.2 ± 82.6	344.8 ± 71.5	0.599
LA HU	358.5 ± 96.8	363.6 ± 97.2	351.6 ± 96.2	0.265
LAA/LA HU	1.00 ± 0.36	0.97 ± 0.26	1.04 ± 0.47	0.065

*LAA* left atrial appendage, *LA* left atrium

All continuous variables are displayed as mean values ± standard deviation. All volumes are displayed in milliliters (mL) and all CT attenuation numbers are displayed in Hounsfield units (HU)

**Table 3:** Volumetric and CT attenuation data in patients with CVA following PVI compared with patients without an observed event

CT Characteristics	Post-PVI CVA	No CVA after PVI	*p*-value
	(*n* = 4)	(*n* = 328)	
Age	58 ± 11	55 ± 13	0.655
Age ≥ 75 years	0 (0%)	13 (4.0%)	0.682
Male gender	4 (100%)	274 (83.5%)	0.373
HTN	2 (50%)	198 (60.4%)	0.674
CHF	0 (0%)	22 (6.7%)	0.589
DM2	0 (0%)	48 (14.6%)	0.407
Vascular disease	1 (25%)	139 (42.4%)	0.484
Prior CVA/TIA	1 (25%)	15 (4.6%)	0.059
CHA_2_DS_2_Vasc > 1	1 (25%)	162 (49.4%)	0.332
CHA_2_DS_2_Vasc, median (IQR)	1 (0, 2.5)	1 (1, 3)	0.543
Anticoagulation			
Warfarin	3 (75%)	198 (60.4%)	0.555
NOAC	1 (25%)	87 (26.5%)	0.952
Aspirin	0 (0%)	43 (13.1%)	0.435
Pre-procedure INR*	2.1 ± 0.3	2.2 ± 0.5	0.792
Rhythm prior to PVI			
Sinus rhythm	1 (25%)	162 (49.4%)	0.332
Atrial Arrhythmia	2 (50%)	164 (50%)	1.000
Paced	1 (25%)	2 (0.6%)	<0.001
LAA volume	13.9 ± 5.3	13.8 ± 6.0	0.989
LA volume	102.6 ± 18.3	99.5 ± 33.8	0.852
LAA/LA volume	0.13 ± 0.04	0.15 ± 0.06	0.655
LAA HU	372.4 ± 92.6	341.9 ± 77.8	0.438
LA HU	410.6 ± 78.8	357.8 ± 97.0	0.279
LAA/LA HU	0.90 ± 0.08	1.00 ± 0.36	0.594

*LAA* left atrial appendage, *LA* left atrium, *CVA* cerebral vascular accident/stroke, *PVI* pulmonary vein isolation

All continuous variables are displayed as mean values ± standard deviation. All volumes are displayed in milliliters (mL) and all CT attenuation numbers are displayed in Hounsfield units (HU)

*INR only includes patients treated with coumadin prior to PVI

The prevalence of CW, WS, CF and CS morphologies were 57.2, 25, 13.2 and 4.5%, respectively amongst post-PVI patients in this population (Table 4). LAA volumes in patients with CS morphology (9.5 ± 3.4 mL) differed significantly from CW (13.6 ± 5.6 mL), WS (14.7 ± 6.4 mL), and CF (14.7 ± 6.6 mL) morphologies (*p* = 0.011). Other volumetric data did not differ between the groups. Additionally, CVA risk factors and CHA_2_DS_2_Vasc scores > 1 were similar amongst the four LAA morphologies. Of note, in this population with a low pre-PVI prevalence of prior CVA, CW morphology was not associated with lower observed rates of prior CVA.

**Table 4:** Baseline demographic data, volumetric and CT attenuation data, and outcomes amongst all 4 LAA morphologies

	Chicken Wing	Cactus	Windsock	Cauliflower	*p*-value
	(*n* = 190)	(*n* = 15)	(*n* = 83)	(*n* = 44)	
LAA volume	13.6 ± 5.6	9.5 ± 3.4	14.7 ± 6.4	14.7 ± 6.6	0.011
LA volume	99.8 ± 34.5	101.8 ± 47.8	98.4 ± 29.3	99.7 ± 33.2	0.982
Total volume	145.2 ± 41.7	144.7 ± 53.2	145.5 ± 38.4	146.8 ± 40.5	0.996
Age	54 ± 13	54 ± 9	56 ± 12	56 ± 16	0.682
Age ≥ 75 years	5 (2.6%)	0 (0%)	5 (6%)	3 (6.8%)	0.65
Male gender	147 (77.4%)	13 (86.7%)	77 (92.8%)	41 (93.2%)	0.954
HTN	115 (60.5%)	10 (66.7%)	53 (63.9%)	22 (50%)	0.45
CHF	14 (7.4%)	0 (0%)	4 (4.8%)	4 (9.1%)	0.549
DM2	26 (13.7%)	1 (6.7%)	16 (19.3%)	5 (11.4%)	0.436
Vascular disease	79 (41.6%)	6 (40%)	35 (42.2%)	20 (45.5%)	0.969
CHA_2_DS_2_Vasc > 1	86 (45.3%)	6 (40%)	48 (57.8%)	22 (50%)	0.246
Prior CVA/TIA	9 (4.7%)	0 (0%)	3 (3.6%)	4 (9.1%)	0.428
Post-PVI CVA	4 (2.1%)	0 (0%)	0 (0%)	0 (0%)	0.388

*LAA* left atrial appendage, *LA* left atrium, *HTN* hypertension, *CHF* congestive heart failure, *DM2* diabetes mellitus, type 2, *CVA* cerebral vascular accident/stroke, *TIA* transient ischemic attack, *PVI* pulmonary vein isolation

All volumes are displayed in milliliters (mL) and all CT attenuation numbers are displayed in Hounsfield units (HU)

## Discussion

We observed an increased incidence of post-PVI CVA in patients with CW morphology on CCTA, however CW morphology was not found to be an independent risk factor for post-PVI CVA after adjusting for known CVA risk factors. Additionally, we did not find any other volumetric or CT attenuation features of the LAA or LA on pre-PVI CCTA that predicted post-PVI CVA. The overall incidence of post-PVI CVA was low in our population (1.2%), as was the prevalence of patients with prior history of CVA prior to PVI (4.7%). These data report on the largest cohort of post-PVI patients to date on NOACs (30.4%) in the lowest estimated CVA risk based on median CHA_2_DS_2_Vasc score.

The applications of CCTA, particularly as an adjunctive modality in various EP procedures, have increased greatly over the past decade. CCTA provides high-resolution, 3-dimensional datasets that can be integrated into electroanatomic mapping systems utilized during PVI 5. CCTA is also a complimentary modality in the evaluation of LAA thrombus and has excellent sensitivity and negative predictive power for excluding thrombus in appropriately protocoled acquisitions [7–9]. Finally, CCTA may serve an important role in the evaluation of the LAA in patients referred for percutaneous LAA closure [12–14]. Prior to the widespread availability and use of CCTA in the EP realm, the LAA was described as a hooked tubular structure with a variable number of lobes. Distinct morphologic features of LAA were first described in a population of patients undergoing PVI in which LAA morphology was evaluated as a risk factor for a prior history of CVA [10]. The morphologies described were cactus (CS), cauliflower (CF), chicken wing (CW) and windsock (WS) [10]. In this initial analysis, patients with CW morphology were nearly 80% less likely to report a prior CVA (OR 0.21, 95% CI 0.05-0.91, *p* = 0.036). When compared to CW morphology, there was an observed 8-fold increased risk of CVA in patients with a CF morphology (*p* = 0.056). Subsequent studies made similar observations regarding the CW morphology as potentially protective with respect to CVA risk while more complex morphologies (i.e. CS) portended increased CVA risk [15–17]. These findings were codified with the publication of a meta-analysis, which included over 2,500 patients from eight studies [18]. Of note, 84% of patients included in this analysis were at low overall CVA risk as defined by a CHADS2 score < 2. CVA risk was lowest amongst patients with CW morphology when compared to non-CW patients (OR 0.46, 95% CI 0.36–0.58, *p* < 0.001) [18]. Our data demonstrated similar results with the majority of prior CVA/TIA events being identified in patients with a CF morphology. The low total incidence of historical CVA/TIA in our patient population likely explains why this difference did not reach statistical significance.

Data evaluating the impact of LAA morphology on post-PVI CVA is less robust. A single-center analysis of 2,570 consecutive patients undergoing PVI reported on 17 thromboembolic events (TE) within 30 days of PVI and compared these patients to 68 propensity-matched patients without peri-procedural TEs in a 1:4 ratio [19]. These authors observed a higher incidence of the CW morphology amongst patients with TEs compared with the matched control patients (65% vs 21%). Of note, the median CHA_2_DS_2_Vasc score was 3 in both groups, the median INR at the time of procedure was <2.0 in both groups, and there was a numerically higher rate of peri-procedural bridging with heparin in the TE group (82% vs 69%, *p* = 0.278) [19]. In contrast, data on 2,069 patients enrolled in a German AF ablation registry found no difference in TE risk between the different LAA morphology groups [20]. TEs were observed in 15 patients (0.7% of the total population) during a 3.078 year follow-up period [20]. The patient population in this analysis was very similar in that the median CHA_2_DS_2_Vasc score was 3 and the vast majority of patients were treated with warfarin. While INR values were not reported in the event-free patients, mean discharge INR in the TE group was 1.5 ± 0.5 and 2.37 ± 0.75 at the time of event. Of note, very small proportions (5%) of patients in this analysis were treated with NOACs for TE prevention.

Our findings of a higher observed rate of CVA amongst patients with CW morphology compared with non-CW morphology is generally in agreement with previously published studies. While we did not find CW morphology to be an independent risk factor, we believe our analysis provides incremental data on the role LAA anatomy may play on CVA/TE risk following PVI for several reasons. The observed incidence of CVA in our population was numerically higher (1.2%) but on par with previous data in the setting of lower median CHA_2_DS_2_Vasc scores. Additionally, NOACs were utilized in 30.4% of patients in the peri-PVI period. Thus, these data may be important as these agents continue to be used more frequently by electrophysiologists around the time of PVI. Finally, given this analysis was performed in a single-payer managed healthcare system where patients have ready access to warfarin or NOACs at no direct cost, compliance with anticoagulation therapy may be higher. Thus, despite low estimated CVA risk and readily available access to anticoagulant medications, there was a numerically higher incidence of CVA in this population suggesting that additional factors beyond the traditional CHA_2_DS_2_Vasc factors and adequate anticoagulation may be important in identifying at-risk patients. Further studies utilizing a multi-modality imaging approach to TE/CVA risk assessment following PVI may be warranted in order to determine whether morphology, when added to other proposed risk factors such as LAA orifice size, degree of LAA trabeculations, LA ejection fraction (EF), LAA peak velocity, and LAA EF, may identify high post-PVI risk patients with low CHA_2_DS_2_Vasc scores [17, 21, 22].

### Limitation

This analysis is limited by its retrospective design and the fact that the patient population is derived from a single-center, tertiary care facility. Overall event rates for post-PVI CVA and prior CVA were low, likely reflecting the relatively low CVA risk of the population based on CHA_2_DS_2_Vasc score, high compliance, and easy access to appropriate anticoagulant therapies. We were unable to determine post-PVI recurrence rates of atrial dysrhythmia, which represents an additional risk factor to CVA in this population. Procedural duration, as a surrogate for the extent of ablation, was not routinely reported in the EMR, thus any association between post-procedural outcomes and the extent of ablation could not be investigated. Additionally, we did not perform an analysis of intra- and inter-observer variability, thus it is possible that LAA morphologies were incorrectly allocated. Previously published data has reported very good agreement in distinguishing CW from non-CW morphology by experienced readers in high-volume centers by CCT (kappa = 0.96, *p* < 0.001) [10]. Applicability of these findings to a general population and comparison to prior studies in this field is limited given the predominately male cohort, younger median age, and the variable post-PVI anticoagulants utilized.

## Conclusions

The CW morphology was observed more commonly in patients who experienced post-PVI CVA. After adjusting for CVA risk factors, CW morphology was not an independent predictor of post-PVI CVA. These findings should be interpreted in the setting of a low CVA event rate amongst a low risk population that was highly compliant with indicated anticoagulation therapy.

## Abbreviations

ACT: Activated clotting time
AF: Atrial fibrillation
CCTA: Cardiac computed tomography angiography
CF: Cauliflower
CI: Confidence intervals
CS: Cactus
CVA: Cerebrovascular accident
CW: Chicken wing
EF: Ejection fraction
EMR: Electronic medical record
EP: Electrophysiology
HU: Hounsfield units
ICE: Intracardiac echocardiography
INR: International normalized ratio
IRB: Institutional review board
LA: Left atrium
LAA: Left atrial appendage
NOAC: Novel oral anticoagulant
OR: Odds ratio
PV: Pulmonary vein
PVI: Pulmonary vein isolation
ROC: Receiver operator characteristics
TE: Thromboembolic
WS: Windsock
